# Pituitary gigantism due to a novel *AIP* germline splice-site variant

**DOI:** 10.1530/EO-24-0003

**Published:** 2024-09-24

**Authors:** Elisa Lamback, Renan Lyra Miranda, Leila Chimelli, Felipe Andreiuolo, Leandro Kasuki, Luiz Eduardo Wildemberg, Mônica R Gadelha

**Affiliations:** 1 Neuroendocrinology Research Center, Endocrinology Section, Medical School and Hospital Universitário Clementino Fraga Filho, Universidade Federal do Rio de Janeiro, Brazil; 2 Neuropathology and Molecular Genetics Laboratory, Instituto Estadual do Cérebro Paulo Niemeyer, Secretaria Estadual de Saúde, Rio de Janeiro, Brazil; 3 Neuroendocrine Unit, Instituto Estadual do Cérebro Paulo Niemeyer, Secretaria Estadual de Saúde, Rio de Janeiro, Brazil; 4 Endocrinology Division, Hospital Federal de Bonsucesso, Rio de Janeiro, Brazil

**Keywords:** AIP, gigantism, pasireotide, SST2, SST5

## Abstract

Pituitary gigantism is a rare pediatric disorder caused by excess growth hormone (GH) secretion. In almost 50% of cases, a genetic cause can be identified, with pathogenic variants in the aryl hydrocarbon receptor-interacting protein (*AIP*) gene being the most common. We present a case of an 11-year-old boy who exhibited progressive vision loss, associated with accelerated linear growth, and weight gain. On physical examination, he had enlarged hands, right eye amaurosis, and was already above his target height. Increased GH and IGF-I concentrations confirmed the diagnosis of pituitary gigantism. Magnetic resonance imaging showed a giant sellar lesion with supra- and para-sellar extensions. He underwent two surgeries which did not achieve a cure or visual improvement. Histopathological analysis revealed a sparsely granulated tumor, negative for somatostatin receptor type 2 (SST2) and an immunoreactivity score of 6 for somatostatin receptor type 5 (SST5). Our published artificial intelligence prediction model predicted an 83% chance of not responding to first-generation somatostatin receptor ligands. Pasireotide was therefore prescribed, and afterward cabergoline was added on. IGF-I concentrations decreased but did not normalize. We discovered a novel germline single nucleotide variant in the splicing donor region of intron 2 of the *AIP* gene (NM_003977.4:c.279+1 G>A), classified as likely pathogenic according to the American College of Medical Genetics and Genomics guidelines.

## Learning points

- Young-onset* AIP-*related pituitary tumors are associated with invasive, large, and treatment-resistant somatotroph tumors.
- Intronic splice variants can lead to truncated proteins, possibly explaining the clinical findings.
- Pasireotide biochemical response depends upon the presence of SST (SST2 and SST5) and possibly post-receptor mechanisms, such as AIP.

## Background

Gigantism is a rare pediatric disorder caused by excess growth hormone (GH). In almost 50% of patients, a genetic cause can be identified, mainly familial isolated pituitary adenoma caused by the aryl hydrocarbon receptor-interacting protein (*AIP*) variants, and X-linked acro-gigantism (X-LAG) or McCune Albright syndrome (Rostomyan *et al.* 2015). Over 100 germline variants have been reported in the *AIP* gene, with less than 20 splice site variants described (Caimari *et al.* 2018). We report a novel germline splice-site variant in *AIP* in a young boy with gigantism.

## Case presentation

**Figure 1:**
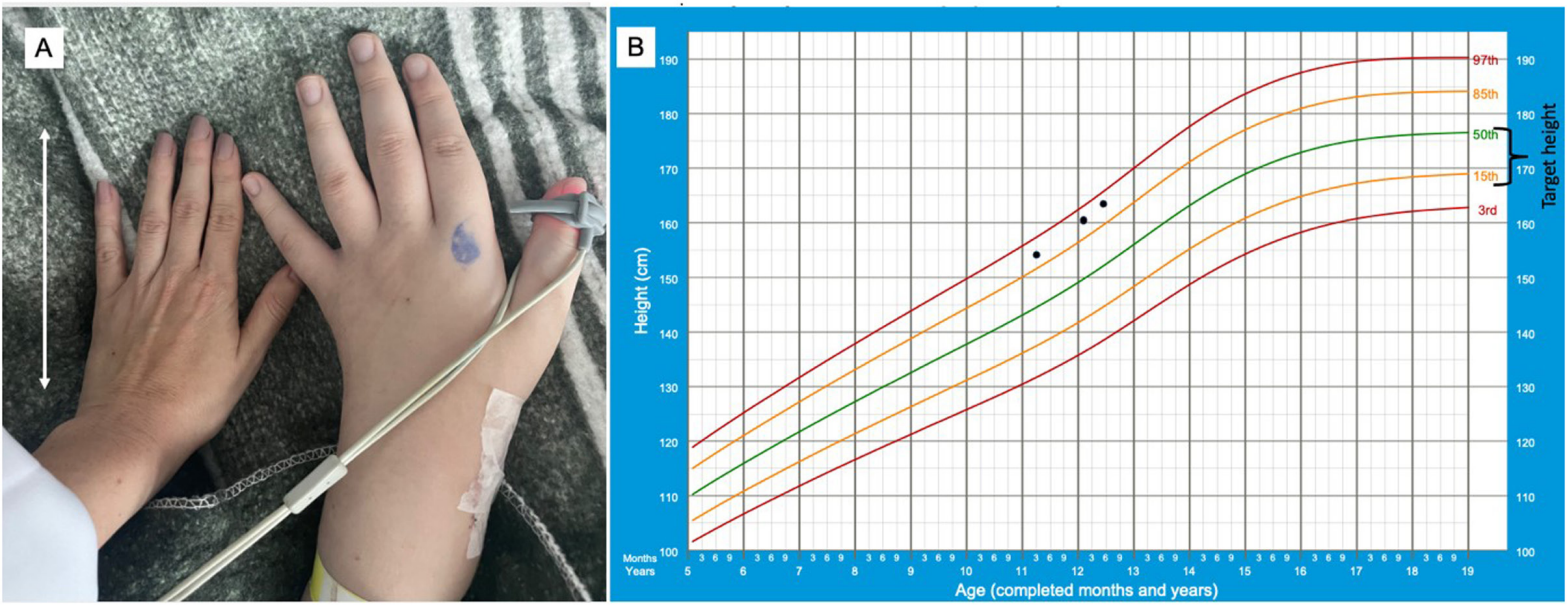
Physical findings. (A) Photograph of the patient’s enlarged left hand compared to Dr Lamback’s hand (adult female) that measures 17 cm (arrow). (B) Growth chart showing the patient’s height (dot) and target height.

An 11-year-old boy presented with progressive vision loss for at least 8 months, accelerated linear growth, and weight gain. On physical examination, he was above his target height (154.5 cm (p85-97), target height of 171.7 cm/p23.6), weight 66 kg (p10-p25), with enlarged hands, Tanner stage G2P3 (Fig. 1).

**Figure 2:**
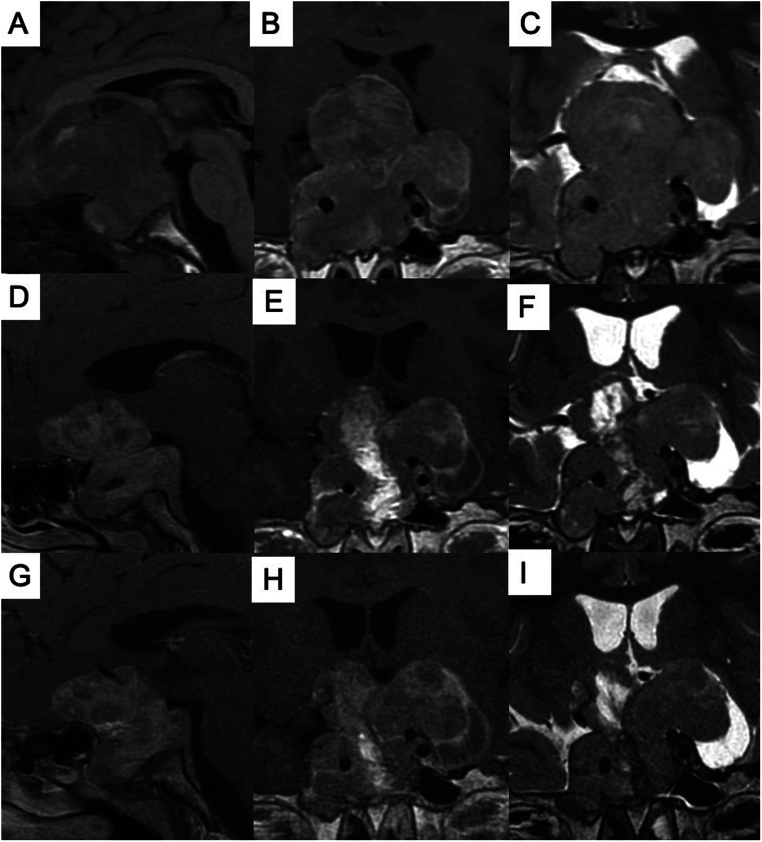
Magnetic resonance imaging. Sagittal T1 post-contrast (A), coronal T1 post-contrast (B), and coronal T2 (C) imaging prior to surgery demonstrate a 5.6 × 4.7 × 5.8 cm (transverse × anterior-posterior × craniocaudal) invasive sellar lesion (Knosp 4) with suprasellar extension, compressing the optic chiasm, isointense on T2 and with heterogeneous contrast enhancement, suggestive of cystic or necrotic degeneration. Sagittal T1 post-contrast (D), coronal T1 post-contrast (E), and coronal T2 (F) imaging after the two surgeries exhibit partial tumor resection. Sagittal T1 post-contrast (G), coronal T1 post-contrast (H), and coronal T2 (I) after 5 months of pasireotide and 2 months of cabergoline show no change in signal intensities or tumor shrinkage. The fat graft in the middle of the tumor reabsorbed partially.

Increased GH of 32.7 ng/mL and insulin-like growth factor I (IGF-I) concentrations of 901 ng/mL (reference range 69–316) confirmed the diagnosis of gigantism. A visual field revealed right eye amaurosis and loss of superior quadrants in the left eye. Sellar magnetic resonance imaging showed a large and invasive tumor (Fig. 2).

## Investigation

**Figure 3:**
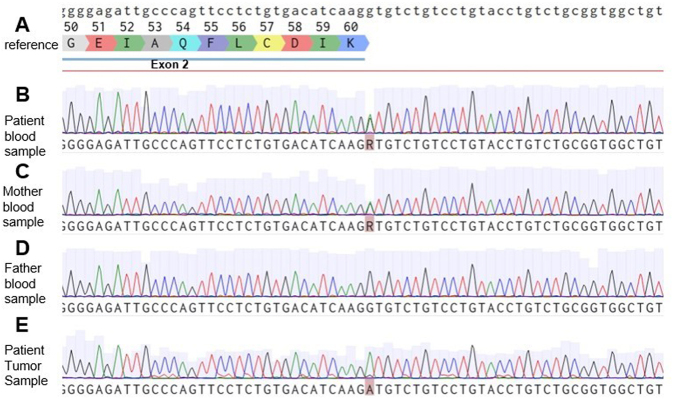
Germline *AIP* sequencing. (A) Reference sequence NG_008969. (B) The patient’s blood sample sequencing exhibiting a single nucleotide variant in the splicing donor region of intron 2 with a nucleotide substitution of G>A in position 279 + 1. (C) The mother’s sequencing demonstrating the same variant. (D) The father was wild type. (E) The patient’s tumor sample sequencing demonstrating LOH.

All exons and splicing sites of *AIP* were sequenced using Sanger sequencing in blood leukocytes. We encountered a novel single nucleotide variant (SNV) in the splicing donor region of intron 2: NM_003977.4:c.279+1 G>A (Fig. 3). Sequencing of the patient’s tumor DNA showed loss of heterozygosity (LOH).

The parents’ sequencing showed that the 48-year-old asymptomatic mother also had the same variant. The patient’s mother has normal IGF-I and monomeric prolactin serum concentrations. The family does not have any known history of pituitary disease, tall stature, or infertility. The patient has no siblings from the mother’s part of the family.

Using FATHMM-XF (Rogers *et al.* 2018) and Eigen bioinformatics tools, the variant was considered pathogenic (non-coding scores of 0.991 and 0.913, respectively). Considering the American College of Medical Genetics and Genomics (ACMG) guidelines, it is considered likely pathogenic (one very strong and one moderate pathogenic criterion (PVS1 and PM2)).

**Figure 4:**
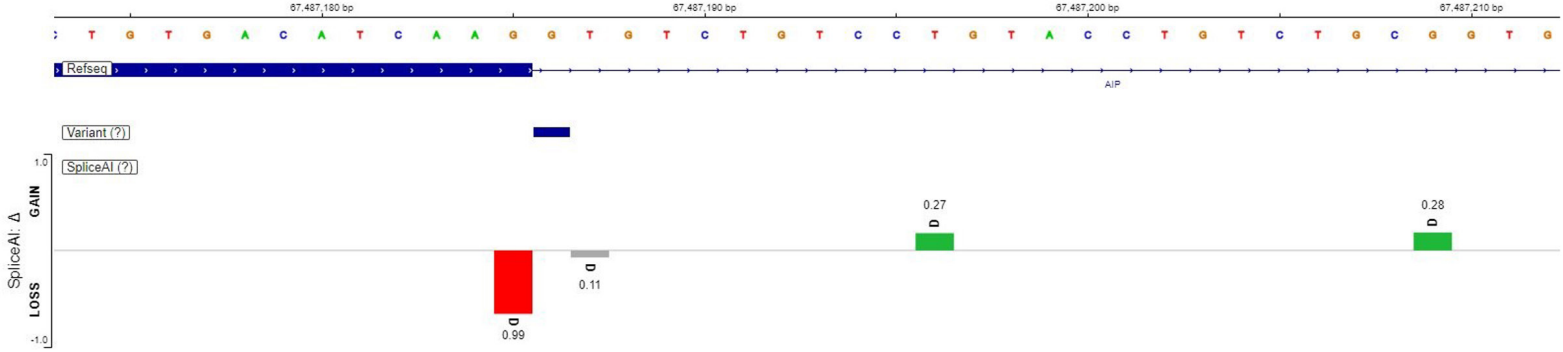
Effects of variant NM_003977.4:c.279+1 G>A on splice-donor region. The presence of the variant results in the loss of the canonical splice site (red column, SpliceAI ΔScore = −0.99) and increases the chance of activating two possible cryptic splice sites (green columns, ΔScore = 0.27 and ΔScore = 0.28).

We performed *in silico* prediction using SpliceAI (Jaganathan *et al.* 2019). The SNV results in the loss of the canonical splice-donor site of exon 2 (SpliceAI ΔScore = −0.99) and can possibly activate one of two nearby cryptic splice sites (CSS) (Fig. 4). The first CSS occurs at position NM_003977.4:c.279+11 (ΔScore = 0.27) and leads to an insertion of 11 bases and a frameshift. The second CSS occurs at position NM_003977.4:c.279+24 (ΔScore = 0.28) and results in an insertion of 24 bases from intron 2 (8 amino acids inserted in the final protein). A ΔScore of 0.20–0.35 has a validation rate of 20–40%, and a ΔScore 0.80–1.00 has a validation rate >80%, indicating that changes with higher scores are more likely to occur and cause impact.

Polymerase chain reaction from complementary DNA of the *AIP* RNA from the tumor sample was performed and showed several different sequences: i) we were still able to identify an *AIP* mRNA equivalent to the NM_003977.4 reference sequence; ii) we confirmed the predicted NM_003977.4:c.279+24 CSS; iii) we identified a 2-base insertion from intron 2 that results in a frameshift and would produce a truncated protein; iv) a smaller sequence that is more expressed than all others and lacks exon 2 and 3; and v) a sequence that lacks exon 2 (Supplementary Materials, see the section on supplementary materials given at the end of this article).

## Treatment

**Figure 5:**
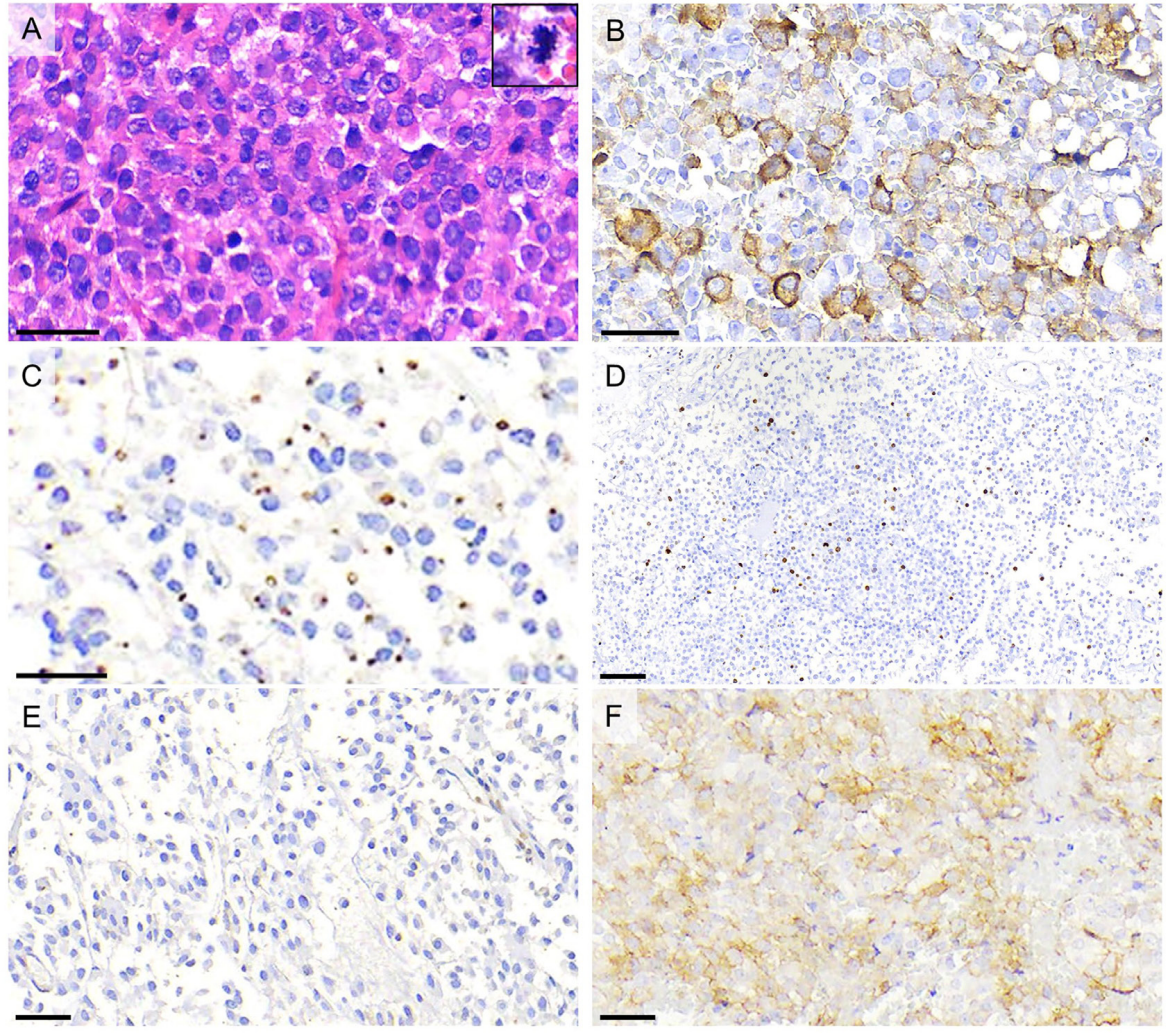
Main histological findings: (A) Hematoxylin and eosin-stained section showing monomorphic tumor cells, displaying round to oval nuclei with conspicuous nucleoli and eosinophilic cytoplasm. A mitotic figure is shown in the insert in the upper right of the panel (up to three mitoses were found in ten 0.237 square mm high-power fields). (B) Immunohistochemistry for growth hormone depicts cytoplasmic positivity in part of tumor cells. (C) CAM5.2 immunostaining highlights cytoplasmic fibrous bodies in tumor cells, characterizing a sparsely granulated tumor. (D) The proliferation index assessed semi-quantitatively (Ki-67 immunostaining; MIB-1 clone) was around 4% overall. (E) Somatostatin receptor type 2 (SST2; UMB1 clone) immunostaining shows no expression in tumor cells. (F) Somatostatin receptor type 5 (SST5; UMB4 clone) immunostaining shows moderate expression in around 60% of tumor cells, corresponding to an immunoreactivity score (IRS) of 6 (reference range: 0–12) (Gatto *et al.* 2013). Scale bars equal 30 micrometers in A and B, and 60 micrometers in D to F.

He underwent transcranial and transsphenoidal surgeries which did not achieve a cure or visual improvement. Gross total resection was not expected as the patient had a very invasive tumor (Knosp 4). Unfortunately, not even vision was improved. The histopathological report is shown in Fig. 5. The diagnosis of a sparsely granulated tumor was made, with negative SST2, moderate expression in around 30% for SST3 (immunoreactive score; IRS 4), and moderate expression in around 60% for SST5 (IRS 6). Prolactin was positive in only sparse cells (around 3%) and was considered negative. This percentage of positive cells was not sufficient to consider the adenoma as a co-secreting tumor (Dottermusch *et al.* 2024). Our artificial intelligence model predicted an 83% chance of not responding to first-generation somatostatin receptor ligand (fg-SRL) (Wildemberg *et al.* 2021). Intramuscular pasireotide was started at a dose of 60 mg every 28 days, with IGF-I concentrations falling >20%, but not normalizing (before: GH 5.0 ng/mL, IGF-I 736ng/mL (69-316); 4 months after pasireotide: GH 12.9 ng/mL, IGF-I 575ng/mL (143-506)). Oral cabergoline was added at a dose of 0.5 mg 3x/week, without IGF-I normalization (1 month after cabergoline: GH 12.3 ng/mL; IGF-I 595ng/mL (143-506)).

## Outcome and follow-up

The patient developed pre-diabetes with pasireotide (glucose 121 mg/dL, glycated hemoglobin A1C 6.1%). He gained 9.0 cm in the last 13 months (present height 163.5 cm at the age of 12 years and 5 months – Fig. 1) and the tumor did not exhibit signal intensity change or shrinkage – Fig. 2. The patient is undergoing radiotherapy and will receive pegvisomant. His treatment will include pasireotide combined with pegvisomant.

## Discussion

The *AIP* variant reported has no registry in the Clinical Genome Resource or ClinVar database. SNV in splice sites can cause different effects on mRNA, and in our case, we could predict two possible outcomes: a frameshift that would result in a stop codon in codon 159 at the end of exon 3 and a truncated protein, or an insertion of 24 bases. Splice variants leading to truncating variations in *AIP* have been considered disease-causing (Boguslawska & Korbonits 2021). Also, since the predicted scores of the new splice-donor sites were weak (<0.40) we have to consider that the SNV could lead to more complex alterations during splicing and whole or multiple exon skipping.

Patients with pathogenic variants in *AIP* present early onset of symptoms, are more often male, with aggressive tumors, requiring multimodal therapy (Marques *et al.* 2020, Korbonits *et al.* 2024), as seen in our patient. Poor response to fg-SRL has been described (Daly *et al.* 2010). In our case, since the response to fg-SRL was very unlikely because of negative SST2 expression and isointensity on T2, we chose not to use it.

Pasireotide was our initial choice. In three cases, pasireotide normalized IGF-I in patients resistant to fg-SRL with *AIP*-mutations (Rostomyan *et al.* 2017, Daly *et al.* 2019). However, in ours and in another case, normalization of IGF-I was not seen (van Santen *et al.* 2021), illustrating that *AIP* variants might be involved in treatment resistance. Pegvisomant may be an alternative; however, *AIP*-mutated tumors are often more aggressive, and pegvisomant has no tumoral effect (Giustina *et al.* 2017). However, in cases like ours, both IGF-I control and tumor size control can be achieved with combined pegvisomant and SRL (Coopmans *et al.* 2022). In conclusion, we describe a novel germline splice variant in *AIP* leading to gigantism. The patient had a sparsely granulated tumor, with a modest response to pasireotide.

## Supplementary Materials

Supplementary Materials

## Declaration of interest

EL has received speaker fees from Ipsen. LK has received speaker fees from Ipsen and Novo Nordisk. LEW has received speaker fees from Ipsen and Recordati, and is sub-investigator in clinical trials from Recordati and Crinetics. MRG has received speaker fees from Recordati, Ipsen and Novo Nordisk, has served as a member of the advisory board of Recordati, Ipsen, Novo Nordisk and Crinetics, and as principal investigator in clinical trials from Recordati and Crinetics. The other authors report no conflicts of interest.

## Funding

This work received a grant (308488/2019-9) from Conselho Nacional de Desenvolvimento Cientifico e Tecnologico (CNPq) to MRG.

## Patient consent

Written informed consent was obtained from the patient’s mother for publication of the submitted article and accompanying images.

## Author contribution and acknowledgements

EL gathered the data and wrote the initial draft, RLM did the Sanger sequencing and pathogenic variant analysis, FA and LC were responsible for the histological slides, LEW is the patient’s main physician and, with LK and MRG, critically reviewed the draft. All authors accepted the final version of the draft.
