# Supplementary material for: Pituitary gigantism due to a novel AIP germline splice-site variant
Source: Endocr Oncol. 2024 Sep 24;4(1):e240003. doi: 10.1530/EO-24-0003 (PMC11466259; doi:10.1530/EO-24-0003)
Supplement: Supplementary Materials [file supplementary_material.pdf]

Supplementary Materials

Methods

For DNA sequencing, we used five primer pairs to cover the six exons of *AIP* (supplementary table 1). To sequence the cDNA and have a broader view of possible effects of the splice variant, we used two different primers pairs. One primer pair comprised a forward primer on exon 1 and a reverse primer on exon 3, the other primer pair comprised a forward primer on exon 1 and a reverse primer on exon 5 (supplementary table 2). All PCR products were evaluated on 3% agarose gel before preparing sequencing reactions, and for cDNA products we purified bands with different sizes with the kit Wizard® SV Gel and PCR Clean-Up System (Promega). It is important to note that since all primers have M13 universal sequences added to them, the final products have additional 36bp. All alignments were performed using Benchling 2024 (https://benchling.com), DNA reference NG_008969 and for RNA (cDNA) NM_003977.4

Supplementary table 1: Primer pairs used for *AIP* DNA sequencing

Target Region	Amplicon Size (no M13)	Sense	**M13**+ Primer Sequence
Exon 1	495bp	Forward	**TGTAAAACGACGGCCAGT**CCGAGACATTCCTAGGCTCCG
		Reverse	**CAGGAAACAGCTATGACC**GCCCGAATTCACCCCCTACTTAAA
Exon 2	381bp	Forward	**TGTAAAACGACGGCCAGT**GGAAGCCCCGTCCCTTATGC
		Reverse	**CAGGAAACAGCTATGACC**AGTCTAGCAGAGGGTGGAGGGAG
Exon 3	492bp	Forward	**TGTAAAACGACGGCCAGT**CGGAGTAGGGTCCCAGTTGTC
		Reverse	**CAGGAAACAGCTATGACC**GGAGACCCAGGGTACTGCCAA
Exon 4 and 5	763bp	Forward	**TGTAAAACGACGGCCAGT**CCAGATGTGGGTCAGGTCTGC
		Reverse	**CAGGAAACAGCTATGACC**TCATGTCTCCTGGCACCATGGG
Exon 6	509bp	Forward	**TGTAAAACGACGGCCAGT**GTGGCATCCTCAGGTCAGGGA
		Reverse	**CAGGAAACAGCTATGACC**GTACCAGGAATGCCAGGTGATGAC

Supplementary table 2: Primer pairs used for *AIP* cDNA sequencing

Target Region	Amplicon Size	Sense	**M13** + Primer Sequence
Exon1-Exon5	689bp	Forward	**TGTAAAACGACGGCCAGT**AGAGCTCCCGGACTTTCA
		Reverse	**CAGGAAACAGCTATGACC**GGTCCAGCACCTCGTAGTA
Exon1-Exon3	300bp	Forward	**TGTAAAACGACGGCCAGT**GGGAGGACGGGATCCAAA
		Reverse	**CAGGAAACAGCTATGACC**CGATGTTGCGGAGACTCTTG

Results

We observed that the exon 1-5 pair generated products of different sizes and we were able to identify: 1- had a size similar to the intended product (689bp + 36bp of M13); 2- around 650bp; 3- around 550bp; 4- around 350bp (Supplementary figure 1).

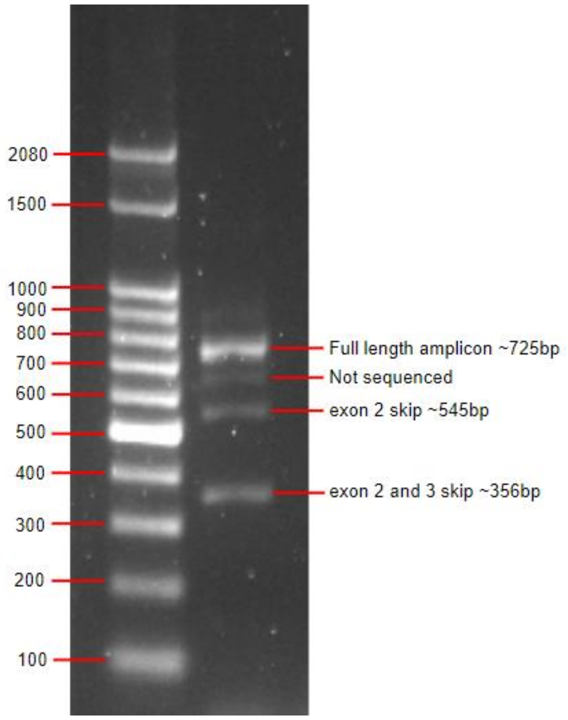

Supplementary Figure 1: AIP cDNA of patient with SNV NM_003977.4:c.279+1. A 100bp DNA ladder on the left and *AIP* cDNA products using primer pair 1-5 on the right.

Since the exon 1-5 primer pair generated a 725bp product when considering M13 sequence, we preferred using the exon 1-3 primer pair to investigate intronic insertions between exons 2 and 3. We found that the electropherogram had double peaks in the beginning of exon 2 in a 66bp region indicating there were 2 sequences in that region, one of them matched the start of exon 3 and the reverse primer with M13 sequence (Supplementary figure 2).

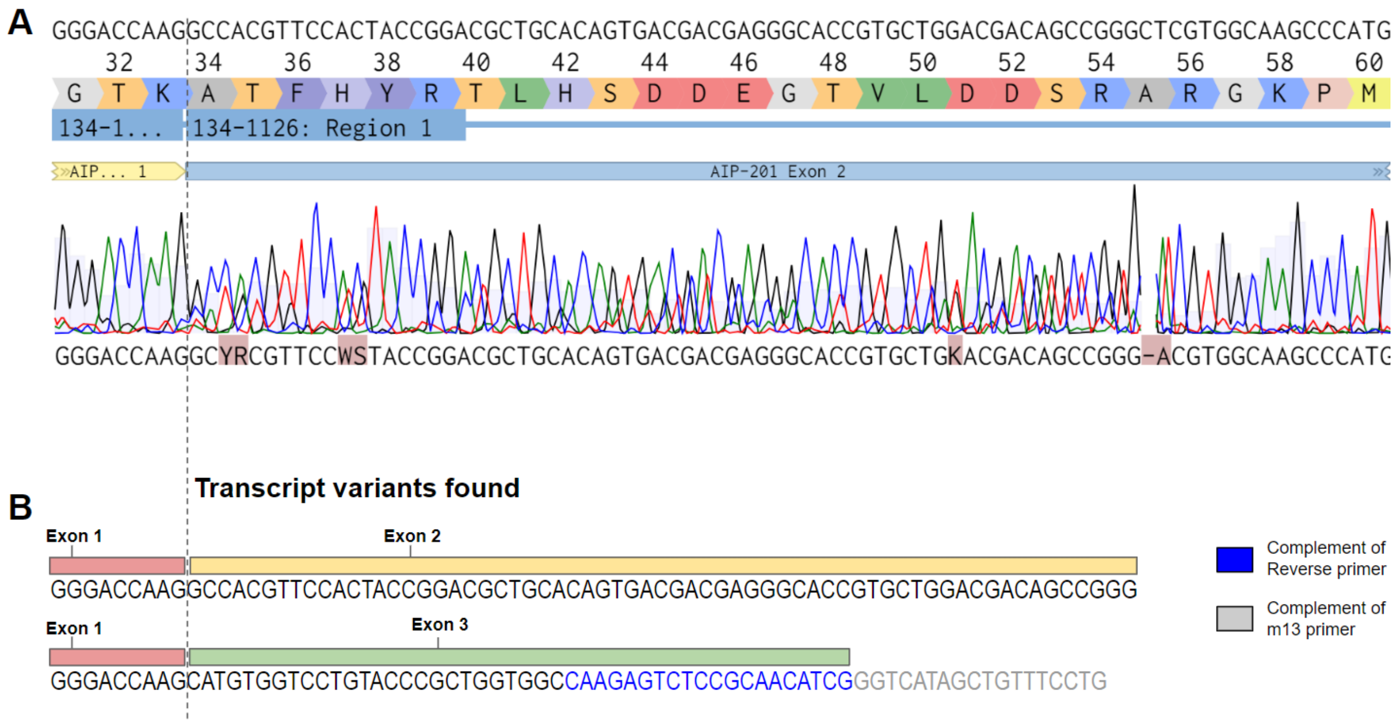

Supplementary Figure 2 Alignment of AIP cDNA sequence around exon 1 and 2 junction from patient. (A) In the start of exon 2 of *AIP,* there is the presence of double peaks on the electropherogram that lasts for 66 bases before going back to single peaks. (B) This 66 bp region is compatible with the start of *AIP*'s exon 3 and we are able to identify M13 complement of the reverse primer, which indicates the presence an AIP cDNA that lacks exon 2. Highlighted sequences: Blue= complement of reverse primer; Gray= complement of reverse M13 sequence.

We found that the electropherogram had double and triple peaks in the cDNA region after exon 2, as expected. Further analysis of the sequencing revealed that the electropherogram had double and triple peaks in the cDNA region after exon 2, as expected by having different products caused by the SNV in the splice donor region. We had the reference sequence present, even with the loss of the canonical splice site, a sequence that had a 24bp intronic insertion and another with a 2bp insertion that lead to frameshift and an early stop codon (Supplementary figure 3).

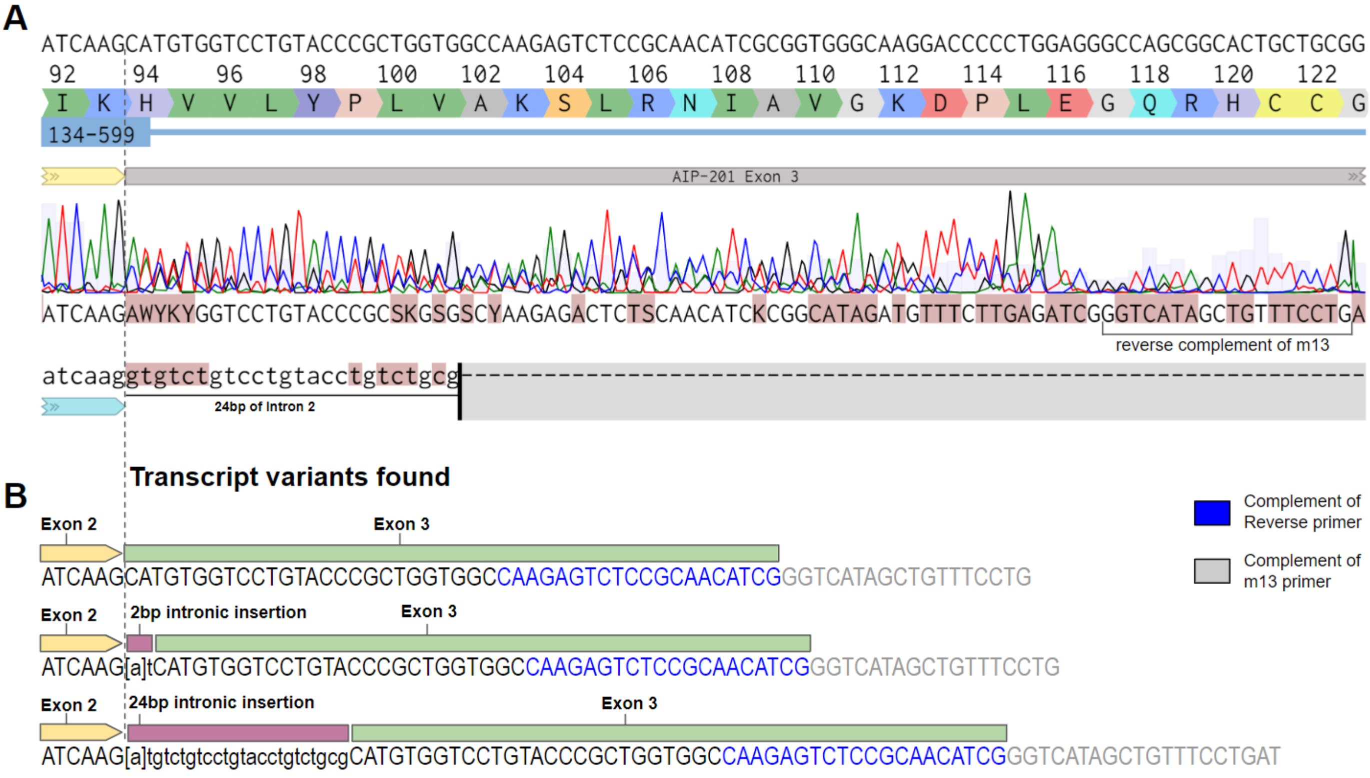

Supplementary Figure 3 **Alignment of AIP cDNA sequence around exon 2 and 3 junction from patient.** (A) In the start of exon 3 of *AIP,* there is the presence of double and triple peaks on the electropherogram. (B) Comparing the peaks to *AIP* reference cDNA NM_003977.4 we identified 3 different sequences: the reference sequence; a 2bp intronic insertion; a 24bp intronic insertion. Highlighted sequences: Blue= complement of reverse primer; Gray= complement of reverse M13 sequence.

We then purified the smaller PCR band from the agarose gel and sequenced it to obtain a sequence of *AIP* cDNA that lacked exon 2 and 3. For alignment, we used the reference ENST00000682659 from Ensembl database (https://www.ensembl.org/), that is an *AIP* transcript that lacks exons 2 and 3.

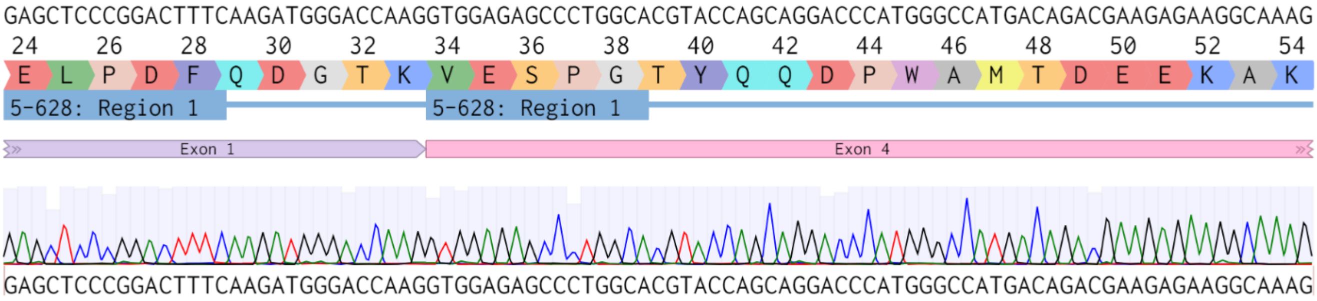

Supplementary Figure 4 Aligment of AIP cDNA product that lacks exon 2 and 3. The alignment was performed between the purified product of ~350bp with the reference ENST00000682659, that is and *AIP* transcript that misses exon 2 and 3.
